# The Ocular Gene Delivery Landscape

**DOI:** 10.3390/biom11081135

**Published:** 2021-08-01

**Authors:** Bhubanananda Sahu, Isha Chug, Hemant Khanna

**Affiliations:** Department of Ophthalmology & Visual Sciences, UMass Medical School, Worcester, MA 01655, USA; bhubanananda.sahu@umassmed.edu (B.S.); isha.chug@umassmed.edu (I.C.)

**Keywords:** retina, eye, gene therapy

## Abstract

The eye is at the forefront of developing therapies for genetic diseases. With the FDA approval of the first gene-therapy drug for a form of congenital blindness, numerous studies have been initiated to develop gene therapies for other forms of eye diseases. These examinations have revealed new information about the benefits as well as restrictions to using drug-delivery routes to the different parts of the eye. In this article, we will discuss a brief history of gene therapy and its importance to the eye and ocular delivery landscape that is currently being investigated, and provide insights into their advantages and disadvantages. Efficient delivery routes and vehicle are crucial for an effective, safe, and longer-lasting therapy.

## 1. Introduction

The complexity of eyes has perplexed scientists of the likes of Charles Darwin. The eye is considered one of the greatest leaps in evolution. Fossil evidence has revealed that eyes appeared ~500 million years ago and became an indispensable tool for survival [1].

The human eye is a camera-type sense organ that allows external visual cues to be transmitted to the brain. The light enters through the anterior chamber of the eye and passes through the cornea, aqueous humor, and the lens before entering the vitreous humor and traversing the inner retina to reach the retina in the posterior compartment. Here, the light signal is converted into an electrical impulse that communicates with the inner retinal neurons and eventually transports to the optic nerve. The signal is then sent to the processing centers in the central nervous system [2].

The vertebrate retina is a light-sensitive tissue containing five major types of neurons (photoreceptors, bipolar cells, amacrine cells, horizontal cells, and retinal ganglion cells) and three types of glial cells (Muller glia, microglia, and astroglia) organized in three distinct layers of cell bodies separated by two synaptic layers. The photoreceptors (rods and cones) account for >70% of the cell types in the retina and are the first responders to light. They contain the photopigment opsin that isomerizes in response to light and generates action potential. The rods are sensitive to lower-intensity light and help us see in starlight (at night). Cones, on the other hand, respond to brighter light and help us see during the day. Commensurately, we depend upon our cones for our day-to-day activities. The importance of cones in maintaining our quality of life is also exemplified by the presence of a cone-rich and rodless central area in the primate retina called the fovea. This structure is part of the macula, which contains the highest density of rods and cones in the central region [2].

Given the importance of visual input for human survival, vision disabilities are one of the top ten disabilities in humans. According to the Centers for Disease Control and Prevention, >3 million people in the United States have vision impairment. By 2050, this number is expected to double to ~6 million people (https://www.cdc.gov/visionhealth/risk/burden.htm; accessed on 6 July 2021). Although the most prevalent eye disorders include complex genetic diseases such as age-related macular degeneration, diabetic retinopathy, cataracts, and glaucoma, the rare forms of inherited retinal degenerations (IRDs) have presented unique challenges in management and treatment.

## 2. Gene Therapy

Gene therapy is a promising technology for treating genetic diseases. The concept of using gene transfer into host cells to treat an underlying genetic condition arose in the 1960s and was later revolutionized by recombinant DNA technology and genetic engineering [3]. One of the first reports of human gene therapy was the use of Shope papilloma virus into three siblings with arginase deficiency. This study was based upon the observation that the virus contained an arginase gene [4,5]. Although there was no effect on the arginase levels in the patients, the general idea of gene transfer appeared sound and realistic. In a span of 3 decades after this experiment, a human gene-therapy trial was performed in a 4-year-old girl with severe combined immunodeficiency (SCID). W. French Anderson and colleagues introduced the wild-type adenosine deaminase (ADA) gene into the patient [6]. Although the therapeutic outcome was short-lived, the excitement in the field of gene therapy for human genetic diseases continued to grow. The field suffered a setback when a patient died because of gene therapy for ornithine transcarbamylase deficiency due to a massive immune response [7]. However, continuing research to improve gene-delivery strategies has generated considerable success and confidence in the field of human gene therapy.

### 2.1. Gene Therapy for Retinal Diseases

There are over 300 genes associated with IRDs, which lead to the dysfunction and degeneration of photoreceptors and/or the retinal pigment epithelium (RPE) [8]. These diseases are inherited in autosomal dominant, autosomal recessive, and X-linked manners. In addition, polygenic and mitochondrial inheritance is reported for IRDs. Furthermore, there is immense clinical heterogeneity associated with IRDs. The patients can present a spectrum of clinical manifestations ranging from congenital or juvenile-onset diseases (such as Leber congenital amaurosis; LCA and Stargardt Disease) to adulthood onset diseases, such as some forms of retinitis pigmentosa, rod-cone dystrophy and cone-rod dystrophy [9,10,11]. Retinal degeneration due to photoreceptor dysfunction is also commonly observed in autosomal recessive syndromic disorders, such as Usher Syndrome, Bardet–Biedl Syndrome, Senior-Loken Syndrome, and Joubert Syndrome [12,13]. Successful gene delivery for IRDs will assist in mitigating the adverse effects of these blinding disorders and improve the quality of life for patients.

### 2.2. Retinal Gene Delivery Route

**Figure 1:**
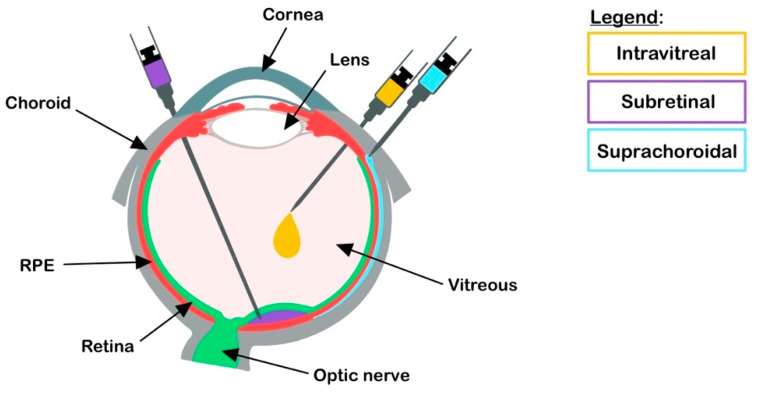
Drug delivery routes. Ocular delivery routes at the indicated locations are depicted. RPE: retinal pigment epithelium.

The retina can be accessed via three distinct routes: intravitreal, subretinal, and suprachoroidal (Figure 1) [14]. The choice of the routes depends upon the target cell type.

The subretinal injection is an invasive surgical procedure in which the therapeutics are delivered between the photoreceptors and the RPE [15]. This vitro-retinal technique requires an operating room, is usually performed under general anesthesia, and carries the risk of retinal tears, detachments, and macular holes. Intravitreal injections (IVIs) on the other hand, are relatively safer and can be performed in the doctor’s office [15]. It is currently used clinically for injecting anti-angiogenic agents for age-related macular degeneration and diabetic retinopathy. Thus, IVI is a preferable procedure for ocular injections. However, IVIs of molecules that are not secreted have poor transduction of the outer retina due to the presence of a physical barrier of the inner limiting membrane and the vitreous. As discussed later in this article, such limitations are being worked on by modifying the delivery vehicle [16] or by using agents to temporarily disrupt the barrier for efficient transduction of the outer retina [17]. Nonetheless, species-specific differences present a bigger challenge. Some approaches that show promising outcomes in rodent models do not hold in larger species, such as sheep, pigs, and non-human primates.

Suprachoroidal injections are a recent breakthrough in the retinal gene-delivery landscape [18]. These are less invasive than subretinal injection and involve accessing the retina by injecting into the space between the choroid (overlaying the RPE) and the sclera [19]. This method has been successful in large-animal models and was demonstrated to be a safer approach in a phase 3 clinical trial to treat uveitis with macular edema [20,21]. Some disadvantages of this method include the use of specialized needles, inaccessibility in smaller animals and difficulty of the AAV vectors to traverse the choroid layer to reach the RPE and the photoreceptors. Nonetheless, the suprachoroidal delivery route provides a unique opportunity to perform less invasive surgeries for retinal gene delivery.

### 2.3. Vectors for Gene Delivery

**Figure 2:**
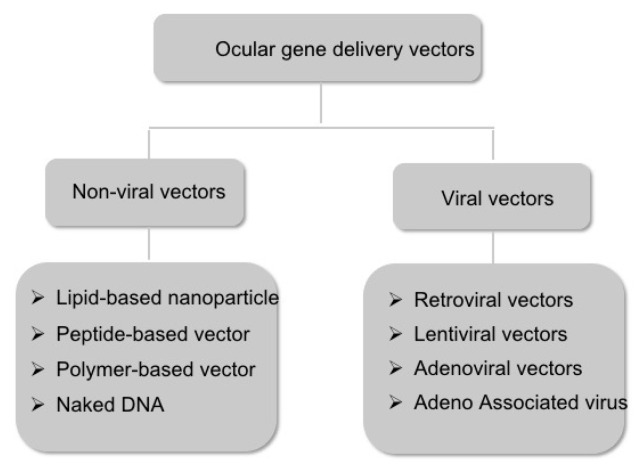
Schematic representation of the major subtypes of the ocular gene delivery vectors.

Vectors for gene therapy are vehicles that carry the gene of interest to the host cells. There are two major subclasses of vectors: nonviral and viral vectors (Figure 2). We will discuss these vehicles in the next sections.

#### 2.3.1. Non-Viral Vectors

Non-viral gene delivery involves delivering a circular, double-stranded plasmid DNA encoding the gene of interest directly into the target cell type [22]. The nanoparticles (NPs) that are used for such delivery can accommodate large sizes of the plasmid DNA, are relatively safer and less immunogenic, maintain long-term protein expression, and carry no risk of insertional mutagenesis [23]. The other advantage associated with NPs is low production cost [22]. The three major criteria for selecting or synthesizing the optimal NP are: cellular uptake, NP composition, and plasmid design.

NPs are usually engulfed by the target cells via phagocytosis or endocytosis. In the eye, the RPE shows both phagocytic and endocytic capacities [24,25]. In addition to phagocytosing photoreceptor outer segments in vivo, RPE can take up large naked DNA or DNA NPs by phagocytosis [26,27]. Photoreceptors, on the other hand, are predominantly endocytic [28,29]. The NPs that undergo clathrin-mediated endocytosis can end up in endosomes, which mature to endolysosomes and undergo degradation. However, caveolin-mediated endocytosis encapsulates the NPs in caveolae vesicles that enter early endosomes or endoplasmic reticulum and escape degradation [30].

NP uptake by target cells also depends upon their composition and net charge. The major types of NPs that are being tested for ocular delivery are as follows:

a. Lipid-based NPs: The naked DNA is further packaged into synthetic compounds to improve DNA transfection efficiency and stability. Lipid-based NPs are composed of a cationic lipid (with a positive charge, a hydrophilic head, and a hydrophobic tail such as DOTAP) and a helper lipid (such as cholesterol) [31]. The positively charged head binds to a negatively charged phosphate group in the DNA to form a compact structure of lipoplexes [32]. When DNA is enclosed in lipoplexes, it is protected from degradation. The lipid-DNA complex enters the cell by endocytosis. Several liposome formulations have been tested for DNA delivery to ocular tissues using intravitreal and subretinal routes [33]. Although ganglion cells and RPE were transfected with high efficiency, the photoreceptors did not exhibit successful DNA transfection [34]. However, it was not until recently that non-viral gene transfer through liposomes could achieve tissue or cell type-specific sustained expression. Liposomes-protamine-DNA (LPD), in combination with a nuclear localization signal (NLS) peptide and a transactivator of transcription (TAT) peptide, make cell-specific and efficient gene delivery with sustained gene expression [35]. Lipid-based compaction of DNA using multifunctional lipids, such as (1-aminoethyl) N-oleicylcysteinyl-1-amino-ethyl) propionamide (ECO), results in a smart escape from endosomal lysis in the cytoplasm and trafficking to the nucleus. ECO nano lipids consist of an ethylenediamine (E) head group, two cysteine (C) functional linkers, and two oleoyl (O) lipophilic tails integrated into DNA for efficient gene delivery to the retina. Sun et al. showed that ECO can efficiently deliver the RPE65 gene into the retinal pigmented epithelium in the Leber congenital amaurosis (LCA) model of Rpe65^−/−^ mice to restore vision [36].

b. Peptide-based NPs: Peptides are used in compaction of DNA for gene delivery and can be considered the best non-viral gene therapy modality. A cationic peptide, enriched in lysine/arginine, makes a tight, compact structure with the DNA. This has an advantage over other non-viral vectors, as it targets specific cell receptors, disrupts the endosomal membrane, and delivers the cargo to the nucleus. It induces minimal immune response and has the capability to be delivered in higher doses [37].

c. Polymer-based NPs: Along with peptide- and phospholipid-based vectors, polymer-based vectors are also used for the compaction of DNA. In this case, the cationic polymer is mixed with DNA to form nanosized polyplexes. Some examples of polymer-based vectors are polyethylene (PEI), dendrimers, and polyphosphoesters. Polymeric nanoparticles are now gaining importance in gene delivery due to their versatility of structural confirmation, biodegradability, and easy synthesis. Some outstanding synthetic polymers include Poly (L-ornithine), polyethyleneimine, and poly(amidoamine) dendrimers. Some natural polymers are chitosan, dextran, and gelatin [38]. Outstanding studies from Naash and colleagues showed that rod-shaped CK30-PEG (polyethylene glycol)-compacted NPs effectively transfected both RPE and photoreceptors and showed efficacious gene therapy of *Rpe65^−/−^* (retinal pigment epithelium 65; model of LCA) and *Abca4^−/−^* (Stargardt Disease) mice [39,40,41].

d. Naked DNA: In a naked plasmid vector delivery, a clinical-grade plasmid DNA is prepared to transfer the gene to the tissue. Clinical trials have been initiated for non-infectious uveitis (NIU) disease of the eye using naked DNA delivery. NIU is an inflammatory symptom in the eye that develops due to eye injury. Systemic anti-TNF (Tumor Necrosis Factor) administration has been approved for NIU to reduce inflammation. The clinical grade plasmid pEYS606 currently in the clinical trial is electro-transferred to the ciliary muscle of the eye [42,43].

Another criterion for efficient non-viral gene delivery is plasmid design. After the DNA enters the cell, it has to reach the nucleus for transcription initiation. In a dividing cell, nuclear envelope breakage during cell division allows the plasmid DNA to enter the nucleus. However, post-mitotic cells such as photoreceptors present unique challenges to access the nucleus. In such cases, the plasmid DNA is modified by addition of regulatory sequences, including promoters, anti-repressor and epigenetic elements as well as nuclear localization signal. The scaffold matrix attachment region (S/MAR)-containing sequence has been shown to maintain the plasmid in an episomal state and bind to nuclear scaffold proteins [44]. This allows efficient attachment to the nuclear matrix and DNA entry into the nucleus. These plasmid modifications have been shown to be effective in gene delivery in *Rpe65^−/−^*, *Abca4^−/−^* and *Rhodopsin^−/−^* [41,45,46].

#### 2.3.2. Viral Vectors

Viral vectors are the delivery system where the genetic materials are introduced in vivo and in vitro to the cell by replication deficient virus. Recombinant replication-deficient adenoviruses have been used in several clinical manifestations to achieve longer-lasting therapeutic effects. In addition, retroviruses, lentiviruses, and adeno associated virus (AAV) are also being used in gene therapy. Each virus type has unique advantages and limitations to transfer the genetic material into host cells.

a. Retroviral vectors: Retroviruses are enveloped single-stranded positive-sense RNA viruses. They reverse transcribe their RNA into double-stranded DNA, which can integrate into the genome of the host cell [47]. It has broad tropism and low immunogenicity, with a packaging ability of 8 kb DNA. The main advantages of this delivery method are persistent integration and expression of transgene in the dividing cell. Some of the drugs that use retroviral gene delivery include strimvelis for SCID and Yescarta for large B cell lymphoma [48]. The disadvantages of this vector are the random integrations of genes into the host genome that raise the possibility of insertional mutagenesis and oncogene activation. This delivery is not suitable for non-dividing post-mitotic cells, including the retina.

b. Lentiviral vectors: Lentiviruses are single-stranded positive-strand RNA viruses and belong to the retrovirus family. The packaging capacity of this viral vector ranges from 8–9 kb. The main advantage of this viral vector is the persistent gene transfer in the transduced tissue and the preferential integration at the 3′ region of the host gene [49]. This virus inserts the genetic material to both the dividing and non-dividing cells and is suitable for ex vivo application. As the lentivirus can accommodate a larger DNA fragment, some of the retinal diseases caused by mutations in larger genes can be delivered with this viral vector. Currently, the non-pathogenic equine infectious anemia virus (EIAV) is in clinical trials to treat Usher Syndrome [50] and Stargardt disease [51,52]. Moreover, Kymriah is another lentiviral vector-based product in clinical trials for ex vivo gene therapy to treat acute lymphoblastic leukemia [53]. The disadvantages of lentiviruses are similar to those of the retrovirus, as they can integrate to the genome and have limited photoreceptor transduction capability.

c. Adenoviral vectors: Adenoviruses (Ad) are non-enveloped, double-stranded DNA viruses with a packaging ability of 30 to 40 kb DNA [54]. Ads are an attractive delivery system because of their broader tropism, grown as high-titer recombinant viruses present in an episomal state, and their ability to transduce dividing and non-dividing cells [55]. As the transduction of the Ad activates innate immune signaling pathways and stimulates immune cells to secrete pro-inflammatory cytokines for robust adaptive immune response, these properties make the adenoviral vector useful for a vaccine vehicle [56]. As it selectively infects cancer cells and induces the expression of pro-inflammatory cytokines to kill tumor cells, Ad is mainly used for gene therapy for cancer cells. About 18.5% of clinical trials use this vector for gene therapy [57]. A major disadvantage of the adenoviral vector is its lengthy production protocol, risk of infection to off-target cells, and severe immune response. There is a high prevalence of serotypes such as Ad5 in the human population, thus increasing the number of neutralizing antibodies against this virus [58]. Therefore, adenoviral vectors are uncommon for gene therapy in the retina.

d. Adeno Associated Virus (AAV): Adeno associated viruses (AAVs) are small (with an icosahederal capsid of ~26 nm diameter), non-pathogenic, non-enveloped, single-stranded linear DNA-containing viruses. They belong to the family Parvoviridae and genus Dependovirus because they can infect only in the presence of a helper virus [59]. The AAVs were discovered as a satellite virus in the adenovirus (Ad) preparation during an electron microscopic examination by the groups of M. David Hoggan and Robert W. Atchison [60]. As it associates with Ad and needs it for its replication, this virus was named “adeno associated virus” (AAV).

The AAV genome is a 4.7 kb linear DNA containing two open reading frames Rep (replication) and Cap (capsid) flanked by inverted terminal repeats (ITRs) (Figure 2). For the AAV to be used as a gene therapy vector, its genome is engineered by removing all AAV protein-encoding sequences and replacing them with the therapeutic cassette. In this recombinant AAV (rAAV) genome, the cassette is flanked by the ITRs that are needed for genome replication and packaging. The therapeutic payload must be under 5 kb and must include the regulatory elements, such as the promoter and polyadenylation signal [61,62].

The rAAV vector-mediated gene delivery into the retina provided a viable and safer approach to treating the underlying disease. One of the first approaches to show gene transfer in mouse retina used rAAV vectors to deliver a reporter gene by subretinal injection into an adult mouse retina [62]. Since then, AAV-mediated gene delivery into the retina has been used in several proof-of-concept studies to target photoreceptors or the RPE in mice and larger models (such as dogs) [63,64,65]. Notably, one of the studies involving the delivery of the *RPE65* gene into mutant dogs showed therapeutic potential, which subsequently led to the approval of the first gene therapy drug (Luxturna^TM^) by the Food and Drug Administration in 2018 [66]. Additional large-animal-model proof of concept studies, including X-linked RP, are now in clinical trials [67,68].

The capsid determines the cell and tissue tropism of the rAAV. Through capsid development, novel rAAV capsids have been discovered or developed that have new and favorable characteristics. Over the years, the AAV virus capsid was modified to infect diverse cell types in the retina. AAV2 and AAV5 were isolated from humans, while AAV4 and AAV8 were isolated from monkeys. These vectors are now being used in clinical trials for human blinding diseases [48]. AAV8 is highly effective in transducing photoreceptors (PRs) and the retinal pigmented epithelium (RPE) as compared to AAV2 and AAV5 in mice [69]. In non-human primates, AAV8 transduces PRs better than AAV2. The surface exposed tyrosine (Y) residues in the AAV capsid undergo ubiquitination and are followed by proteasome-mediated degradation in the cytoplasm. Mutations of these residues prevent the phosphorylation and subsequent ubiquitination and degradation [70]. A tyrosine residue mutation in the capsid of the AAV8 to phenylalanine AAV8 (Y733F) produces a higher transgene expression than the wild-type AAV8 [71]. Using a rational design approach, novel variants of AAV2 and AAV5 have been generated, which demonstrate improved retinal transduction in non-human primate retina and tissue [72]. Moreover, novel AAV9-based capsids (AAV-PHP.eB) have been designed that can cross the blood-retinal barrier when delivered systemically [73].

The route of delivery can also impact rAAV tropism in the retina due to the presence of anatomical barriers. A majority of rAAV serotypes can transduce the RPE when delivered subretinally. However, their tropism in the rod and cone PRs varies greatly. AAVs targeting the photoreceptors or the RPE poorly penetrate the outer retina after intravitreal injection due to the presence of the inner limiting membrane. Recently, Dalkara et al. reported the generation of a novel capsid AAV7m8, which when administered intravitreally could transduce photoreceptors in the primate fovea and the photoreceptors and RPE of mice [74].

Another cell-type that is challenging to transduce is the ON-bipolar cells in the inner retina. These cells are the second order neurons that transmit the information from the photoreceptors to the ganglion cells. Recent work from the Bennett lab showed the development of a new AAV serotype AAV8BP2 by in vivo directed evolution [75]. This rAAV can target the ON-bipolar cells by subretinal injection in mice. Given the interest in bipolar cells for optogenetic therapies, AAV8BP2 offers an attractive avenue for further studies.

#### 2.3.3. Disadvantages of rAAV Gene Delivery

The prevalence of neutralizing antibodies and immunological response in humans against viral capsids is an important consideration when selecting rAAVs for gene delivery. Although rAAVs are non-integrating and efficiently transduce retinal populations, intraocular inflammation and loss of efficacy have been associated with rAAV-gene delivery [76]. This response was observed in both intravitreal and subretinal delivery routes and is linked to the capsid and the dose of the AAV. AAV activates innate immune response, which releases the inflammatory cytokines and type-1 interferons. Neutralizing antibodies against the capsid can also reduce the therapeutic potential of the gene delivery [76]. To evade the immune system, George Church and colleagues recently reported the generation of engineered AAV vectors that are intrinsically less immunogenic. They achieved this by incorporating immunomodulatory noncoding sequences to “cloak” the vector from immune responses [77].

Additional disadvantages include the limited size of the transgene (<5 kb) that can be packaged into AAV capsids [78]. As a result of these potential limitations, alternative strategies such as dual AAV vector systems and NPs are being developed. The dual AAV vector system involves splitting the large transgene into two <5kb fragments that are packaged and co-delivered into the cell. The DNA fragments are then recombined into a complete functional transgene intracellularly either by homologous recombination or trans-splicing mechanisms [79,80,81].

## 3. Conclusions

Non-viral delivery systems offer distinct advantages, including unlimited payload size, low immunogenicity, and minimal side effects. Although the efficacy of this approach has been reported earlier, such as in *Abca4^−/−^* mice, the non-viral gene delivery strategy has not yet been used in any clinical trials for ocular diseases. Anatomical barriers in the retina and pH sensitivity of the nanoparticles are some of the many environmental challenges for efficient non-viral gene delivery and prolonged gene expression.

Based on the considerable research on the different viral vectors for gene delivery to date, rAAVs seem to be the safest and the most reliable vehicles for gene delivery. Advances in capsid identification, safety, and transduction are needed for their robust, successful, and wider clinical applications. Viral gene therapies are promising tools to transfer genetic material. The insights of precise gene therapy increase the speed for the discovery to restore vision that is destroyed by blinding diseases.

## Data Availability

Not applicable.
